# The Medical Letter Bag

**Published:** 1919-03

**Authors:** E. H. Martin

**Affiliations:** Hot Springs, Ark.


### THE MEDICAL LETTER-BAG

A Department for Short Correspondence from Physicians

"Piece out our imperfections with your thoughts."--Shakespeare

### Will One-Man Practice Stop?

Hot Springs, Ark., February 13, 1919.

Editor Texas Medical Journal:

I think the most important question before the medical profession today is the necessity for getting together and stopping as much as possible, **one-man practice.**

One man, even the good old country doctor, cannot do everything, although we must admit that he goes a long way and is a much better practitioner than the one-man city doctor.

When a patient needs real medical investigation and attention he needs more than one man and, unless he has a lot of money to pay specialists or unless he be a pauper and gets the attention of the whole staff of a hospital, he cannot get it.

The honest bill-paying citizen cannot afford to pay a half-dozen doctors and he will not pose as a pauper, so he gets left. The solution is the getting together of the doctors of the county or the township or the town, and formation of a hospital and within it a laboratory, X-Ray outfit and the specializing of some of these doctors in certain branches.

This tendency is obvious when we consider the Mayo Clinic. It becomes suggestive when we consider some of the large firms in Dallas and other cities where six or seven doctors work together; but it is just as feasible in the country.

If the county authorities will build hospitals the doctors will soon put aside their personal bickerings and join in to do actual team work and, while they may not be gainers personally, the great American public will be.

It is highly probable that this millennial condition will not be universal until the U. S. Government or the State Government takes charge of medical practice and furnishes medical attention to everyone free of charge just as naturally as they send a fire-engine to put out a fire. Then the physicians will be on salaries and such teams and hospitals and clinics will be naturally organized with a certain number of physicians to do emergency work and others to do work in the laboratory and hospital.

There is a whole lot more in this if you will just think it over.

Yours sincerely,

E. H. MARTIN, M. D.

